# Changes in High-Density Lipoproteins Related to Outcomes in Patients with Acute Stroke

**DOI:** 10.3390/jcm9072269

**Published:** 2020-07-17

**Authors:** Lourdes M. Varela, Elena Meseguer, Bertrand Lapergue, David Couret, Pierre Amarenco, Olivier Meilhac

**Affiliations:** 1 Inserm U1148, Paris University, 75018 Paris, France; 2 Instituto de Biomedicina de Sevilla (IBiS)/Hospital Universitario Virgen del Rocío/CSIC/Departamento de Fisiología Médica y Biofísica-Universidad de Sevilla, 41013 Sevilla, Spain; 3 Department of Neurology and Stroke Center, Paris University, 75018 Paris, France; elena.meseguer@aphp.fr (E.M.); pierre.amarenco@aphp.fr (P.A.); 4 Department of Neurology, Stroke Center, Foch Hospital, 92150 Suresnes, France; bertrand.lapergue@gmail.com; 5 CHU de La Réunion, 97410 Saint-Pierre, France; david.couret@chu-reunion.fr; 6 Université de La Réunion, Inserm U1188 DéTROI, F-97490 Sainte-Clotilde, France

**Keywords:** HDL, stroke, functional outcome, PON1, blood-brain barrier (BBB) dysfunction, low-density lipoproteins (LDL) oxidation

## Abstract

Modifications in high-density lipoprotein (HDL) particle sizes and HDL-binding proteins have been reported in stroke patients. We evaluated whether the lipoprotein profile, HDL composition and functionality were altered in stroke patients according to their clinical outcome using the modified Rankin Score at 3 months. Plasma samples were obtained from stroke patients treated with intravenous thrombolysis. Levels of cardiovascular and inflammatory markers in plasma were measured using the Human CVD Panel 1 (Milliplex^®^ MAP). Lipoprotein subfractions from plasma were quantified by non-denaturing acrylamide gel electrophoresis, using the Lipoprint^®^-System (Quantimetrix^®^), and HDLs were isolated by ultracentrifugation. Relative amounts of paraoxonase-1 (PON1) and alpha-1 anti-trypsin (AAT) in the isolated HDLs were determined by Western blot. HDL anti-inflammatory function was evaluated in human blood–brain barrier endothelial cells stimulated with 100 ng/mL TNFα, and HDL antioxidant function was evaluated via their capacity to limit copper-induced low-density lipoprotein oxidation. Stroke patients with unfavorable outcomes had a lower proportion of small-sized HDLs and increased plasma levels of E-selectin (SELE) and the intercellular adhesion molecule 1 (ICAM1). HDLs from patients with unfavorable outcomes had lower levels of PON1 and displayed a blunted capacity to reduce the expression of SELE, interleukin 8 (IL8) and the monocyte chemoattractant protein-1 (MCP1) mRNA induced by TNFα in endothelial cells. These HDLs also had a reduced antioxidant capacity relative to HDLs from healthy donors. In conclusion, an increased ratio of large/small HDLs with impaired anti-inflammatory and antioxidant capacities was associated with unfavorable outcomes in stroke patients. Alteration of HDL functionality was mainly associated with a low amount of PON1 and high amount of AAT.

## 1. Introduction

High-density lipoproteins (HDL) are highly heterogeneous in size, shape and composition [1,2]. HDL-cholesterol concentration remains a biomarker to assess cardiovascular risk. However, it has been suggested that the relationship between HDLs and acute stroke is related to HDL particle size and HDL-binding proteins rather than HDL-cholesterol concentration [3]. HDL subfractions differ in their ability to exert their antiatherogenic functions (cholesterol removal, anti-inflammatory, antioxidant and endothelial cell protection) [4]. Small, dense HDL particles appear to display a higher cholesterol efflux capacity, antioxidant effects and anti-inflammatory properties than large HDL particles [5].

Some of the identified proteins related to HDL functions are paraoxonase-1 (PON1) and alpha-1 anti-trypsin (AAT), which show antioxidant and anti-inflammatory properties, respectively [6,7,8]. PON1 is an enzyme synthesized in the liver and then secreted in blood, where it mainly binds to HDLs. It is considered responsible for many of the antiatherogenic properties of HDLs by reducing low-density lipoproteins (LDL) oxidation, a critical process of atherogenesis [9,10]. AAT is an acute-phase reactive plasma protein that belongs to the family of serine protease inhibitors. It is the main physiological inhibitor of neutrophil elastase, an enzyme produced by activated neutrophils in atherosclerotic lesions, which degrades extracellular matrix components and stimulates the production of pro-inflammatory cytokines that contribute to plaque instability [11,12].

HDLs may participate in the pathology of stroke at different levels [3]. Acute stroke is characterized by an activated endothelium that favors the recruitment of leukocytes. HDLs may exert anti-inflammatory effects on the endothelium but also on leukocytes [13]. In endothelial cells, HDLs inhibit cytokine-induced expression of vascular adhesion molecules, such as the intercellular adhesion molecule 1 (ICAM1) and E-selectin (SELE), and hence reduce leukocyte adhesion and transmigration [14,15]. In leukocytes, HDLs limit the adhesion and transmigration of leukocytes to endothelial cells, mainly through modulation of CD11b expression [16,17]. Interestingly, in a rat model of thromboembolic stroke, we showed that the injection of plasma HDLs after a stroke was associated with a reduced blood–brain barrier (BBB) breakdown and decreased neutrophil recruitment in the infarct area. In this model, stained HDLs were mainly associated with endothelial cells, suggesting that their primary protective effect was on the BBB [18]. After an ischemic stroke, injured brain tissue releases reactive oxygen species and pro-inflammatory cytokines into the extracellular compartment. These mediators activate brain endothelial cells participating in the disruption of the BBB that may cause brain edema, neuronal death and, in some cases, hemorrhagic transformation [19].

Since HDL particles may participate in the pathology of stroke and potentially limit subsequent brain damage, the aim of the present study was to evaluate whether the lipoprotein profile, HDL composition and functionality were altered in stroke patients according to their clinical outcome, determined using the modified Rankin Score at 3 months (mRS). The mRS is the most widely used outcome measure in trials for acute stroke interventions. The mRS is an ordinal scale ranging from 0 (no symptoms at all) to 6 (death), measuring the degree of disability or dependence in everyday life [20].

## 2. Material and Methods

### 2.1. Patient Selection

The study cohort included 50 ischemic stroke patients treated with intravenous thrombolysis at the Bichat Hospital (Paris, France). Before treatment, the patients’ NIHSS (National Institute of Health Stroke Scale) and mRS scores were assessed by certified stroke physicians. We used the dichotomous approach for the mRS: 0–2 for a favorable outcome and 3–6 for an unfavorable outcome. In total, 4 patients with diabetes mellitus were excluded since HDL functionality was reported to be altered under diabetic conditions [21,22]. Control HDL particles were isolated from the blood of healthy volunteers. Exclusion criteria for controls were a history of cardiovascular, renal or hepatic disease, diabetes mellitus or metabolic syndrome, hypertension and the presence of neurological dysfunction. The control group consisted of 12 healthy subjects of similar age and sex to the patients group (6 men and 6 women, non-smokers, 42–72 years old). This study was conducted in accordance with the Declaration of Helsinki and the protocol was approved by the ethics review board of Bichat Hospital (SC090966). Written informed consent was provided.

### 2.2. Plasma Sampling

Blood from patients was collected in EDTA-containing tubes within 4 h of stroke, immediately processed (2000× *g* for 10 min and 2500× *g* for 15 min at room temperature) and stored at −80 °C until analysis. Samples were collected from January 2009 to June 2013.

### 2.3. Plasma Cytokines and Chemokines Quantification

Plasma levels of specific cytokines and chemokines involved in the inflammatory response were measured using the Human CVD Panel 1 from Milliplex^R^ MAP Kit (Millipore, Burlington, MA, USA). Biomarkers included in this kit were as follows: SELE, VCAM1 (vascular cell adhesion molecule 1), ICAM1, MPO (myeloperoxidase), PAI1 (plasminogen activator inhibitor-1), and MMP9 (matrix metallopeptidase 9). Briefly, 25 µL of standard or assay buffer, 25 µL of matrix solution or sample and 25 µL of beads were added to the well and incubated overnight at 4 °C under gentle shaking. After incubation, the beads were washed twice with wash solution. Next, 25 µL of detection antibodies was added and incubated for 1 h at room temperature. Then, 25 µL of streptavidin-phycoerythrin was added to each well and incubated for 30 min. The plate was washed and 150 µL of sheath fluid was added into each well, and the plate was analyzed using a Luminex 200^TM^ (BioRad, Hercules, CA, USA) with a reporter laser of 532 nm and a photomultiplier tube (PMT) as a reporter detector with a bandwidth of 565 to 585 nm. Incubation times and reader PMT (RP1) setting were optimized for this particular assay panel. In our study, the RP1 PMT was 568.74 Volts and the RP1 Target was 3784.

### 2.4. Lipid Assay

Total cholesterol (TC), HDL and LDL/VLDL (LDL/very-low density lipoproteins) levels were measured enzymatically in plasma samples using the EnzyChrom AF Assay Kit (BioAssay Systems, Hayward, CA, USA), following the manufacturer’s protocol.

Lipoprotein subfraction profiles were assessed using the Lipoprint^®^ LDL & HDL Subfraction Testing System (Quantimetrix, Redondo Beach, CA, USA). This method is based on non-denaturing, linear polyacrylamide gel electrophoresis of lipid stained EDTA plasma, allowing the visualization and quantification of different lipoprotein subfractions by their migration distance. LDLs are separated into large (fractions 1 and 2) and small dense particles (fractions 3 to 7), whereas HDLs are categorized into large, intermediate and small HDL particles. The system includes data analysis software to determine the subfraction concentrations based on their electrophoretic mobility.

### 2.5. HDL Isolation by Ultracentrifugation

HDLs were isolated from 900 μL of plasma by ultracentrifugation. In brief, plasma density was adjusted to 1.22 g/mL with KBr and overlaid with KBr saline solution (*d* = 1.063 g/mL). Ultracentrifugation was performed at 120,000× *g* for 4 h at 10 °C to separate LDL, IDL (intermediate density lipoproteins) and VLDL from the rest of the plasma. The density of the bottom fraction containing HDL was adjusted to 1.25 g/mL with KBr and overlaid with KBr saline solution (*d* = 1.22 g/mL). The second ultracentrifugation was performed at 100,000× *g* for 16 h at 10 °C. After this step, the HDL fraction (top layer of the tube) was recovered as a single band, extensively rinsed with saline and concentrated using a centrifugal concentration device. The purity of the isolated HDLs was confirmed by lipoprotein agarose gel electrophoresis, and the total protein concentration was determined by the BCA assay (Thermo Fisher Scientific, Rockford, Illinois, USA), using bovine serum albumin as a standard.

Apolipoprotein A1 (APOA1) concentration in plasma and HDLs was determined using an ELISA kit from Mabtech AB (Sweden).

### 2.6. Western Blot Analyses of HDL

HDLs (4 µg total proteins) from each patient were subjected to sodium dodecyl sulphate-polyacrylamide gel electrophoresis (SDS-PAGE, 12% acrylamide). For immunoblotting, proteins were transferred to polyvinylidene fluoride membranes which were blocked with 5% (*w*/*v*) low fat milk in Tris-buffer saline (pH 7.4/0.1% Tween-20) and incubated overnight at 4 °C with the respective primary antibody: AAT (dilution 1:5000; abcam, UK), PON1 (dilution 1:500; abcam, Cambridge, UK) and anti-APOA1 (dilution 1:1000; AbD serotec). In all cases, an appropriate horseradish peroxidase-conjugated secondary antibody (Jackson ImmunoResearch, West Grove, PA, USA) was used at a dilution of 1:5000, and bound immunoglobulins were visualized by the enhanced chemiluminiscence technique (Amersham Biosciences, Little Chalfont, UK). Densitometry analysis was performed using the ImageJ software [23].

### 2.7. In Vitro BBB-Functionality Assay

Human brain endothelial cells hCMEC/D3 were cultured in complete endothelial basal medium-2 (EMB + 2.5% of fetal calf serum and supplements containing hydrocortisone and growth factors). HCMEC/D3 cells were grown to confluence in 12-well plates and were made quiescent by 12 h incubation in serum-deprived medium before stimulation with 100 ng/mL of TNFα (Millipore, Burlington, MA, USA) ± 0.3 mg/mL of HDLs for 8 h.

### 2.8. RNA Isolation/RT-PCR Analysis

Total RNA was extracted from endothelial cells using TRIzol Reagent (Invitrogen, Carlsbad, CA, USA), as instructed by the manufacturer. RNA quality was assessed on the basis of the A260:280 ratio in a NanoDrop ND-1000 Spectrophotometer (Thermo Scientific, UK). RNA (1 μg) was subjected to reverse transcription at 42 °C for 30 min using the iScript cDNA synthesis kit (BioRad, Hercules, CA, USA). Expression levels of mRNA for specific genes were measured by RT-PCR in the CFX96 system (BioRad, Hercules, CA, USA). For each PCR reaction, one-tenth of the cDNA template was added to the SYBR Green PCR Master Mix (Sigma-Aldrich, St. Louis, MO, USA) containing the primer pairs for *MCP1* (monocyte chemoattractant protein-1) and *IL8* (interleukin 8) (chemokines involved in the recruitment of leukocytes), for *VCAM1* and *SELE*, (adhesion molecules), for the barrier-constituting junctional protein *CLDN1* (claudin 1) and for *GAPDH* used as a housekeeping gene, as determined by RefGen [24] as the most stable control gene in a set of 4 control genes (*GAPDH, HPRT, 18S* and *RPL13*). *GAPDH* presented a mean gene stability value of 1.9, compared to 4 for the least stable control gene (*18S*). Primer sets were designed using the Primer3 Input software and obtained from Sigma-Aldrich (USA). The primer sequences and MIQE details are provided in Table 1. All amplification reactions were performed in triplicate, and average threshold cycle (Ct) numbers of the triplicates were used to calculate the relative mRNA expression of the candidate genes. The magnitude of change in mRNA expression for candidate genes was calculated using the standard 2^−(ΔΔCt)^ method. All data were normalized to endogenous reference (GAPDH) gene content and expressed as percentage of controls.

### 2.9. Determination of the Capacity of HDLs to Inhibit LDL Oxidation

LDL (100 µg/mL) oxidation was induced by the addition of CuSO_4_ to yield a concentration of 5 µmol/L. HDLs (8 µg/mL) from healthy donors and patients were incubated with LDL to test their capacity to delay oxidation. LDL lipid oxidation to conjugated dienes was monitored every minute for 2 h by measuring the change in absorbance at 234 nm. Using experimental curves of oxidation kinetics, the duration of the lag time, the maximal propagation rate (V_max_) of lipid peroxidation and the maximum conjugated diene formation (T_max_) were calculated. The lag time was defined from the absorbance curve as the time given by the intercept of the two straight lines from the lag and propagation phases on the time axis. V_max_ was determined as the peak of the first derivative of the absorbance curve (change in absorbance as a function of time, dA/dt), and T_max_ was obtained by the projection of V_max_ onto the time axis of the first derivative of the absorbance curve [25].

### 2.10. Statistical Analysis

Analyses were performed using GraphPad Prism 5.0 (USA). Differences between pairs were tested using the Mann–Whitney test. Statistical analysis for group comparisons was performed using the Kruskal–Wallis test, followed by a post hoc Dunn’s comparison test. Statistical analysis for qualitative variables was performed using Fisher’s exact test. Correlation analysis was performed using the Spearman test. Differences were considered statistically significant when *p*-value < 0.05.

## 3. Results

### 3.1. Patient Characteristics

The main characteristics of the patients included in the study are detailed in Table 2.

### 3.2. Plasmatic Parameters

**Figure 1:**
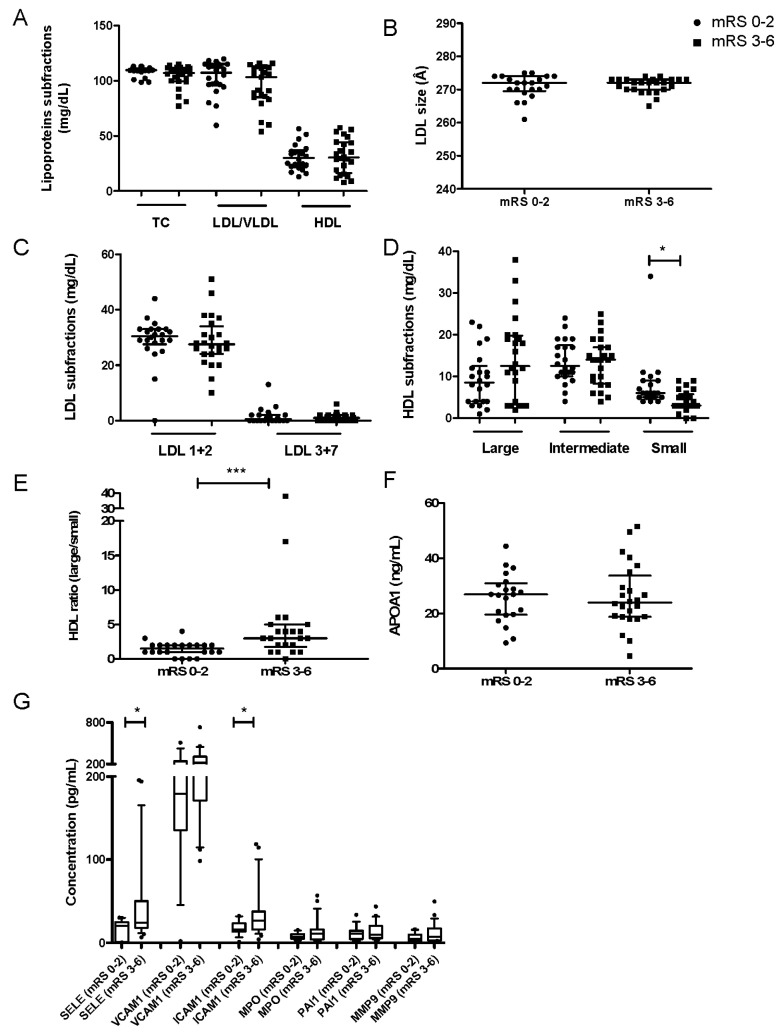
Lipoprotein subfractions, plasma levels of APOA1 and inflammatory cytokines between stroke patients with favorable (mRS 0–2, *n* = 22) or unfavorable (mRS 3–6, *n* = 24) outcomes. (**A**) Concentration of lipoprotein subfractions. (**B**) Mean LDL particle size. (**C**) Concentration of LDL subfractions. (**D**) Concentration of HDL subfractions. (**E**) Ratio of large/small HDLs. (**F**) Concentration of APOA1. Values are medians ± IQR. (**G**) Concentration of each inflammatory marker quantified. Boxes delineate the 10th and 90th percentiles, and points demonstrate outliers. * *p* < 0.05, *** *p* < 0.001. Abbreviations: TC, total cholesterol; LDL/VLDL, low-density lipoprotein/very low-density lipoprotein; HDL, high-density lipoprotein; APOA1, apolipoprotein A1; IQR, interquartile range.

We found no differences in plasma lipids (Figure 1A) in patients with favorable or unfavorable outcomes at 3 months (mRS 0–2 vs. mRS 3–6) for TC (108.60 ± 0.92 vs. 104.0 ± 2.11 mg/dL), LDL/VLDL (102.60 ± 3.30 vs. 95.67 ± 3.95 mg/dL) or HDL-C (30.74 ± 2.48 vs. 31.81 ± 3.19 mg/dL) levels. A comparison of mean LDL size and subfractions revealed no differences between groups (Figure 1B,C). As shown in Figure 1C, the contribution of larger LDL subfractions (LDL 1 + 2) to the total profile was greater in both groups. Patients with a favorable outcome (mRS 0–2) presented a slight increase in large LDL particles (LDL 1 + 2), together with a decrease in the proportion of the small dense LDL subfractions (LDL 3 + 7), but the variation was not statistically significant. Noteworthy, HDL subclass distribution (Figure 1D) revealed that patients with a favorable outcome (mRS 0–2) had a significant increase in the proportion of small-sized HDLs, while the proportion of larger HDL particles was lower, but not significantly different, when compared to patients with an unfavorable outcome (mRS 3–6). Therefore, the ratio of large/small HDLs was significantly lower in these patients (Figure 1E). The circulating levels of APOA1 (Figure 1F) did not differ according to outcome at 3 months (25.66 ± 1.93 in mRS 0–2 vs. 26.02 ± 2.39 ng/mL in mRS 3–6).

Regarding biomarkers associated with the inflammatory response in ischemic stroke (Figure 1G), SELE (45.94 ± 11.02 vs. 16.13 ± 2.57 ng/mL, *p* = 0.019) and ICAM1 (35.19 ± 6.27 vs. 18.12 ± 2.23 mg/dL, *p* = 0.023) levels were significantly higher in patients with unfavorable outcomes (mRS 3–6) as compared to those with favorable outcomes (mRS 0–2). The other assayed biomarkers followed the same trend but did not reach statistical significance: VCAM1 (253.60 ± 30.31 vs. 202.30 ± 32.08 ng/mL), MPO (14.02 ± 2.98 vs. 7.99 ± 1.06 ng/mL), PAI1 (14.30 ± 2.36 vs. 11.27 ± 2.16 ng/mL) and MMP9 (11.78 ± 2.43 vs. 6.07 ± 1.37 ng/mL).

### 3.3. HDL-Associated Proteins

**Figure 2:**
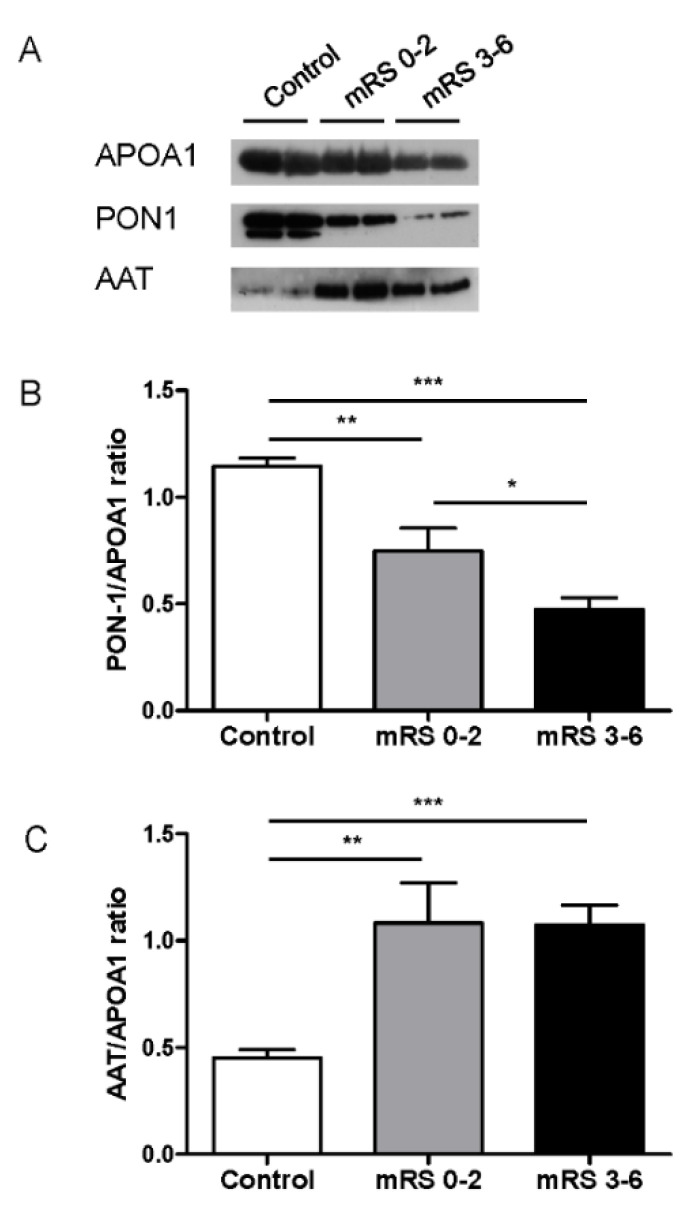
HDL-associated proteins. (**A**) Western blot analysis of AAT, PON-1 and APOA1 in isolated HDLs. (**B**) Quantification of HDL-associated PON1 and (**C**) AAT after normalization to APOA1 intensity in healthy donors (*n* = 12) and stroke patients with favorable (mRS 0–2, *n* = 22) or unfavorable (mRS 3–6, *n* = 24) outcomes. Values are means ± SEM. * *p* < 0.05, ** *p* < 0.01, *** *p* < 0.001. Abbreviations: APOA1, apolipoprotein A1; PON-1, paraoxonase-1; AAT, alpha-1 anti-trypsin.

Protein composition and function of HDLs may differ between patients suffering acute cerebral infarction and healthy subjects [26]. PON1 and AAT have been reported to be important mediators of some anti-inflammatory, antioxidant and potentially atheroprotective effects of HDL [27]. Therefore, we analyzed the abundance of these proteins in HDLs isolated from stroke patients versus healthy donors (Figure 2). HDL-associated PON1 and AAT were evaluated by Western blot analysis and normalized to APOA1 (the major HDL protein), and no differences were found between groups of patients when measured by ELISA (data not shown). Both PON1 and AAT protein levels were significantly different in HDLs from stroke patients compared with HDLs from healthy donors. PON1 protein content was significantly decreased in HDLs in stroke patients (Figure 2A,B), being lower in those with unfavorable outcomes (mRS 3–6) (0.48 ± 0.05 vs. 0.75 ± 0.11 AU, *p* = 0.049). AAT was significantly more abundant in HDLs from stroke patients relative to those of healthy donors (Figure 2A–C); however, no differences were found according to outcome (1.08 ± 0.19 vs. 1.08 ± 0.11 AU, *p* = 0.3).

### 3.4. Correlations

We evaluated the correlations of different lipoproteins subfractions, circulatory inflammatory biomarkers and the HDL-associated proteins measured with their clinical outcome at 3 months (mRS). The results shown in Table 3 demonstrate that the levels of large HDL, the ratio of large/small HDL and circulating SELE were significantly positively correlated with the unfavorable outcomes (0.387, *p* = 0.008; *r* = 0.579, *p* < 0.001; *r* = 0.41, *p* = 0.007, respectively) and that the levels of small HDL and the HDL-associated PON1 were significantly negatively correlated with the unfavorable outcomes (−0.422, *p* = 0.004; −0.391, *p* = 0.007, respectively).

### 3.5. HDL Anti-Inflammatory Activity

**Figure 3:**
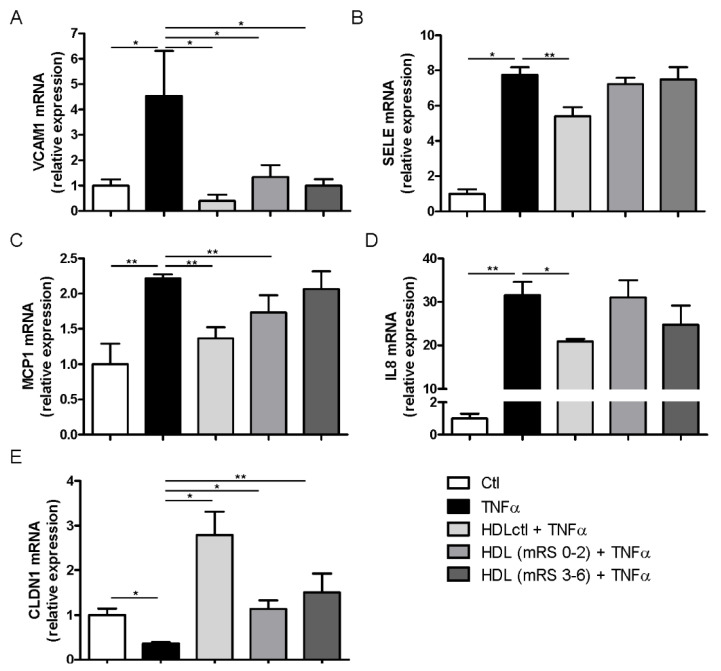
Effects of HDLs on brain–blood barrier (BBB) endothelial cells stimulated by TNFα. mRNA expression of (**A**) *VCAM1*, (**B**) *SELE*, (**C**), *MCP1*, (**D**) *IL8* and (**E**) *CLDN1* in hCMEC/D3 cells co-incubated with TNFα and HDLs from healthy donors (HDLctl, *n* = 6) or stroke patients with favorable (mRS 0–2, *n* = 22) or unfavorable (mRS 3–6, *n* = 21) outcomes. Values are means ± SEM. * *p* < 0.05. Abbreviations: *VCAM1*, vascular cell adhesion molecule 1; *SELE*, E-selectin; *MCP1*, monocyte chemoattractant protein-1; *IL8*, interleukin 8; *CLDN1*, claudin-1.

In order to identify functional changes in the brain endothelium, we assessed the capacity of HDLs to modulate gene expression in response to stimulation by the pro-inflammatory cytokine TNFα (Figure 3). Eight hours of TNFα induction significantly increased gene expression levels of *VCAM1*, *SELE, MCP1* and *IL8*, whereas *CLDN1* levels were decreased compared to untreated cells. It is well established that HDLs have the potential to inhibit vascular endothelial inflammation and to improve endothelial function [28]. Indeed, simultaneous administration of TNFα with HDLs isolated from healthy donors effectively limited the changes in gene expression observed following TNFα stimulation. However, HDLs isolated from stroke patients were less prone to preventing gene changes induced by TNFα. HDLs from stroke patients, independently of their outcome, significantly limited TNFα-induced expression of *VCAM1* (increase) and *CLDN1* (decrease) gene expression (Figure 3A,E). HDLs from patients with favorable outcomes (mRS 0–2) significantly limited TNFα-induced expression of *MCP1*, whereas those from patients with unfavorable outcomes (mRS 3–6) lost this ability (Figure 3C). Finally, HDLs from stroke patients did not limit the increase in *SELE* and *IL8* expression, regardless of the outcome (Figure 3B,D).

### 3.6. Antioxidant Activity of HDL

**Figure 4:**
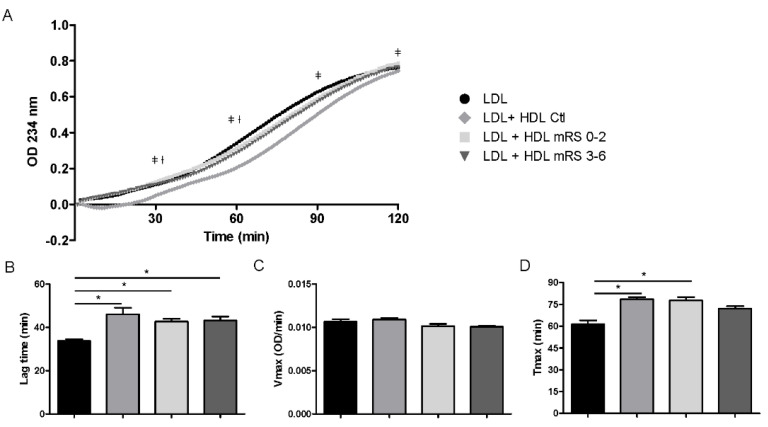
Effects of HDLs on LDL oxidation. (**A**) Curve of conjugated diene formation during oxidation of LDL in the presence of HDL from healthy donors (HDLctl, *n* = 3 pooled samples) or stroke patients with favorable (mRS 0–2; *n* = 7) or unfavorable (mRS 3–6; *n* = 6) outcomes. (**B**) Duration of lag time. (**C**) Maximal propagation rate (V_max_). (**D**) Time of maximum conjugated diene formation (T_max_). Values are means ± SEM. ǂ *p* < 0.05 LDL + HDLctl vs. LDL + HDmRS 3–6; † *p* < 0.05 LDL + HDL mRS 0–2 vs. LDL + HDL mRS 3–6; * *p* < 0.05.

HDLs have been reported to have significant antioxidant activity that is mainly demonstrated via the inhibition of LDL oxidation [7,29]. Hence, we tested the ability of HDLs isolated from patients and healthy donors to prevent copper-induced LDL oxidation. HDLs from healthy donors protected LDLs against oxidation, as evidenced by an increased lag time. HDLs from stroke patients also delayed the initiation of the lag phase, and no difference was observed according to the outcome (Figure 4A,B). We did not observe significant differences for V_max_ between HDLs from stroke patients relative to HDLs from healthy subjects (Figure 4C). However, HDLs from stroke patients with unfavorable outcomes (mRS 3–6) did not modify the time to reach the maximal diene production (T_max_) induced by cooper in LDLs, suggesting a decreased protective effect of HDLs from patients versus heathy subjects (Figure 4D).

## 4. Discussion

In this study, we found that HDLs from patients with unfavorable outcomes at 3 months (mRS 3–6) exhibited an increased ratio of large/small HDLs that presented lower levels of PON1 compared to patients with favorable outcomes (mRS 0–2). In addition, HDLs from patients with unfavorable outcomes displayed a blunted capacity to reduce the expression of *SELE*, *IL8* and *MCP1* induced by TNFα in brain endothelial cells and also had a reduced antioxidant capacity relative to HDLs from healthy donors.

We did not find significant differences in the lipid profile parameters between stroke patients in relation to their outcome. It has been shown that stroke patients had significantly higher levels of TC, LDL-C and HDL-C than controls [30], but no relationship between any of the lipid levels and functional outcome at 3 months had been previously described [31]. Marked differences in LDL size have been found in different studies between stroke patients and controls, suggesting that small dense LDL particles are associated with stroke [32,33]. However, there is little information available on the relationship between LDL particles and functional outcome in stroke. Previously, Song et al. [34] showed an association between small dense LDL particles and poor functional outcome in stroke patients. In our study, we did not find any differences between groups according to LDL size. This might be explained by the method used for the determination of LDL particle size. Song et al. determined the peak LDL particle diameter by electrophoresis, using gradient gel and calibration curves after ultracentrifugation. In our study, we assessed LDL subfractions and the mean LDL particle size by the Lipoprint technology on the whole plasma. The Lipoprint^®^ system analyses all lipoprotein fractions and subfractions in plasma and measures the amount of cholesterol in each LDL and HDL subfraction. Moreover, the LDL subfraction analysis in this system is approved for clinical use. It has been demonstrated that there is a good agreement for the estimation of LDL size distribution (large–small dense LDL) between the Lipoprint system and the polyacrylamide gradient gel electrophoresis. However, absolute LDL particle size may differ between the two methods mentioned [35].

One important finding of our study was the presence of smaller HDL particles in stroke patients with unfavorable outcomes. To the best of our knowledge, this is the first report describing the different distribution of HDL particles in relation to the outcome after a stroke, in patients treated by intravenous thrombolysis. Several studies have shown an inverse relationship between HDL-C levels and cardiovascular risk [36]. However, different HDL subfractions may exhibit distinct protective activities, and emerging evidence suggests that HDL quality (distribution of HDL subfractions and/or functionality) could be more informative than HDL-C levels when predicting cardiovascular risk [37,38,39]. Thus, the main novelty of the present work is the study of the impact of different HDL subfractions on the outcome at 3 months after ischemic stroke. Here, we have focused our attention on the association between stroke patient outcome and both HDL particle size and functionality. In this regard, we observed that patients with unfavorable outcomes presented significantly lower proportions of small HDL particles and higher ratios of large/small HDLs relative to patients with favorable outcomes.

HDL-associated proteins are considered to be a major determinant of HDL biological functions. Small HDL particles have been shown to exert more potent anti-inflammatory and antioxidant effects compared with large HDLs [40,41]. In fact, our previous study demonstrated the abundance of larger HDL particles in stroke patients compared to controls, resulting in the inappropriate protection of brain endothelial cells under oxygen-glucose deprivation conditions [26]. Proteomic studies have reported that HDLs can carry AAT, an anti-elastase and anti-inflammatory protein that may have protective effects against BBB dysfunction [42]. In addition, it has been proposed that the antioxidant properties of HDL rely, at least partially, on the enzyme PON1 [43]. When compared to controls, we found that AAT was more abundant in HDLs from stroke patients compared to healthy controls, but no differences related to the outcome were found. Using an in vitro model of the BBB, it has been reported that HDLs could attenuate the proteolysis and the associated increased permeability of the BBB by inhibiting the elastase-mediated role of activated polymorphonuclear neutrophils [8]. The increased AAT content associated with HDL may reflect a response to the increased activation of neutrophils often reported in stroke [44,45]. We found that HDLs increased the expression of claudin-1, a key mediator of BBB sealing and maintenance [46]. Interestingly, HDLs from stroke patients were less prone to induce claudin-1 expression, regardless of the outcome. In relation to the antioxidant capacity of HDLs, we observed that HDL-associated PON1 was significantly decreased in stroke patients with unfavorable outcomes, which may potentially reduce their ability to protect LDL from oxidation. Previous studies have shown that stroke patients undergo inflammation and oxidative stress, associated with defective HDLs and low PON1 activity [47]. Human PON1 is an enzyme that is highly effective in preventing LDL oxidation, a phenomenon involved in atherogenesis [48]. In addition, it has been shown that inhibition of LDL oxidation prevents the upregulation of MCP1 secretion, providing evidence that PON1 may be able to inhibit atherosclerosis at an early stage [49]. In this regard, our results support the importance of PON1 in both anti-inflammatory and antioxidant activities attributed to HDL atheroprotective functions. In our study, the amount of PON1 in HDL particles may be important in controlling both functions, since low PON1 levels in HDLs were associated with a reduced protection against TNFα-induced BBB production of MCP-1 and a blunted prevention of LDL oxidation.

## 5. Conclusions

Unfavorable outcomes in stroke may be related to the reduced proportion of small HDL particles present in the circulation of stroke patients. A higher ratio of large/small HDL particles is observed in stroke patients with unfavorable outcomes (mRS 3–6). These HDLs have decreased PON1 and increased AAT amounts compared with HDLs isolated from healthy controls and also display lower antioxidative and anti-inflammatory activity.

Appropriately designed studies are required in order to evaluate HDL particle size and associated proteins in stroke patients to consider the supplementation with functional HDLs as a therapeutic approach to improve their functional outcome after a stroke.

## Figures and Tables

**Table 1:** Gene-specific oligonucleotides used for the RT-PCR analysis.

Gene	RefSeq.	Forward Primer (5′ to 3)	Reverse Primer (5′ to 3′)	Annealing Temperature (°C)	Amplicon Size (bp)	Efficiency (%)	R^2^
*VCAM1*	NM_001078	AAGGCAGGCTGTAAAAGAATTG	GTAGACCCTCGCTGGAACAG	60	125	98.6	0.997
*SELE*	NM_000450	AGCCCAGAGCCTTCAGTGTA	AACTGGGATTTGCTGTGTCC	60	244	102.8	0.996
*MCP1*	NM_002982	TCAGCCAGATGCAATCAATG	TCCTGAACCCACTTCTGCTT	60	186	98.1	0.987
*IL8*	NM_000584	AGACAGCAGAGCACACAAGC	ATGGTTCCTTCCGGTGGT	60	62	99.9	0.972
*CLDN1*	NM_021101	CCGTTGGCATGAAGTGTATG	AAGGCAGAGAGAAGCAGCAG	60	233	99.1	0.996
*GAPDH*	NM_002046	GAGTCAACGGATTTGGTCGT	GATCTCGCTCCTGGAAGATG	60	225	99.8	0.998
*HPRT*	NM_000194	ACCCCACGAAGTGTTGGATA	AAGCAGATGGCCACAGAACT	60	248	91.2	0.944
*18S*	NR_145820	AAACGGCTACCACATCCAAG	CCTCCAATGGATCCTCGTTA	60	155	104.8	0.991
*RPL13A*	NM_012423	CCTGGAGGAGAAGAGGAAAGAGA	GAGGACCTCTGTGTATTTGTCAA	60	124	96.6	0.999

Abbreviations: RefSeq, NCBI sequence accession number; bp, base pair; R^2^, correlation coefficient of standard curve; *VCAM1*, vascular cell adhesion molecule 1; *SELE*, E-selectin; *MCP1*, monocyte chemoattractant protein-1; *IL8*, interleukin 8; *CLDN1*, claudin 1; *GAPDH*, glyceraldehyde-3-phosphate dehydrogenase; *HPRT*, hypoxanthine guanine phosphoribosyl transferase; *18S*, 18S RNA ribosomal; *RPL13A*, ribosomal protein L13a.

**Table 2:** Main characteristics of the patients.

	mRS 0–2 (*n* = 22)	mRS 3–6 (*n* = 24)	*p* ^a^
Age, yr, mean (range)	67 (35–94)	75 (42–96)	0.86
Male gender, %(*n*)	64 (14)	50 (12)	0.39
Weight, kg, mean (SD)	75 (15)	70 (22)	0.08
Hypertension, %(*n*)	50 (11)	58 (14)	0.77
Hypercholesterolemia, %(*n*)	32 (7)	42 (10)	0.46
Smoking, %(*n*)			
Never	64 (14)	75 (18)	0.41
Former	14 (3)	13 (3)	
Current	23 (5)	8 (2)	
NIHSS at admission, median (IQR)	8 (9)	16 (7)	<0.001
Systolic BP, mean (SD)	159 (15)	150 (22)	0.15
Diastolic BP, mean (SD)	86 (14)	80 (16)	0.25
Glucose, median (IQR)	116 (34)	132 (38)	0.11
Hemorrhagic transformation, %(*n*)			
None	86 (19)	58 (14)	0.07
Asymptomatic	14 (3)	29 (7)	
Symptomatic	--	13 (3)	
90-day mortality, %(*n*)	--	16 (73)	<0.001

^a^*p*-values computed using Fisher’s exact test for qualitative variables and unpaired *t*-test for continuous variables. Abbreviations: mRS, modified Rankin score, SD, standard deviation; IQR, interquartile range; BP, blood pressure.

**Table 3:** Correlation of lipoprotein subfractions, circulatory inflammatory biomarkers and HDL-associated proteins with the outcomes of patients using the modified Rankin Score at 3 months (mRS).

Variables	Correlation Coefficent (R)	*p*-Value
LDL 1 + 2 (mg/dL)	−0.1289	0.3932
LDL 3 + 7 (mg/dL)	−0.0569	0.7068
Large HDL (mg/dL)	0.3871	0.0079
Intermediate HDL (mg/dL)	0.1360	0.3674
Small HDL (mg/dL)	−0.4217	0.0035
HDL ratio (large/small)	0.5786	<0.0001
Plasmatic APOA1 (ng/dL)	0.0301	0.8425
APOA1 in HDL (ng/dL)	−0.0107	0.9435
Plasmatic SELE (ng/mL)	0.4096	0.0071
Plasmatic VCAM1 (ng/mL)	0.2626	0.1218
Plasmatic ICAM1 (ng/mL)	0.3136	0.0587
Plasmatic MPO (ng/mL)	0.2097	0.2197
Plasmatic tPAI-1 (ng/mL)	0.0554	0.7485
Plasmatic MMP9 (ng/mL)	0.2584	0.1225
PON1/APOA1 ratio in HDL	−0.3912	0.0072
AAT/APOA1 ratio in HDL	0.1880	0.2110

Abbreviations: LDL, low-density lipoprotein; HDL, high-density lipoprotein; APOA1, apolipoprotein A1; PON-1, paraoxonase-1; AAT, alpha-1 anti-trypsin; R, Spearman’s correlation coefficient.

## Data Availability

The data that support the findings of this study are available from the corresponding author upon reasonable request.
